# Pulmonary MRI in Newborns and Children

**DOI:** 10.1002/jmri.29669

**Published:** 2024-12-06

**Authors:** Neil J. Stewart, Nara S. Higano, Lena Wucherpfennig, Simon M.F. Triphan, Amy Simmons, Laurie J. Smith, Mark O. Wielpütz, Jason C. Woods, Jim M. Wild

**Affiliations:** ^1^ POLARIS, Division of Clinical Medicine, School of Medicine & Population Health, Faculty of Health The University of Sheffield Sheffield UK; ^2^ Insigneo Institute of In Silico Medicine, The University of Sheffield Sheffield UK; ^3^ Center for Pulmonary Imaging Research, Pulmonary Medicine and Radiology, Cincinnati Children's Hospital Medical Center Cincinnati Ohio USA; ^4^ Department of Pediatrics University of Cincinnati College of Medicine Cincinnati Ohio USA; ^5^ Department of Radiology Cincinnati Children's Hospital Medical Center Cincinnati Ohio USA; ^6^ Department of Diagnostic and Interventional Radiology University Hospital Heidelberg Heidelberg Germany; ^7^ Translational Lung Research Center Heidelberg (TLRC), German Center for Lung Research (DZL) Heidelberg Germany; ^8^ Department of Diagnostic and Interventional Radiology with Nuclear Medicine Thoraxklinik at University Hospital Heidelberg Heidelberg Germany

**Keywords:** lung, MRI, pediatric, neonatal, free‐breathing, hyperpolarized ^129^Xe

## Abstract

Lung MRI is an important tool in the assessment and monitoring of pediatric and neonatal lung disorders. MRI can provide both similar and complementary image contrast to computed tomography for imaging the lung macrostructure, and beyond this, a number of techniques have been developed for imaging the key functions of the lungs, namely ventilation, perfusion, and gas exchange, through the use of free‐breathing proton and hyperpolarized gas MRI. Here, we review the state‐of‐the‐art in MRI methods that have found utility in pediatric and neonatal lung imaging, the structural and physiological information that can be gleaned from such images, and strategies that have been developed to deal with respiratory (and cardiac) motion, and other technological challenges. The application of lung MRI in neonatal and pediatric lung conditions, in particular bronchopulmonary dysplasia, cystic fibrosis, and asthma, is reviewed, highlighting our collective experiences in the clinical translation of these methods and technology, and the key current and future potential avenues for clinical utility of this methodology.

**Evidence Level:**

2

**Technical Efficacy:**

Stage 2

MRI is becoming increasingly used for imaging the lungs of children and infants with respiratory conditions, where the risks associated with ionizing radiation are of primary concern. In recent years, lung MRI methods have matured, notably: T_1_, T_2_‐weighted and contrast‐enhanced perfusion sequences are used as part of clinical protocols for assessment of lung abnormalities in cystic fibrosis (CF)^1^; ultra‐short echo time (UTE) ^1^H lung MRI now provides comparable quality to CT for structural imaging in fibrotic disease^2^ and nodule detection^3^; inhaled hyperpolarized ^129^Xe MRI for assessment of lung function is used clinically in the UK in patients 5 years and above^4^ and has recently been approved by the FDA for use in patients down to an age of 12 years.^5^ A PubMed search for (MRI) AND ((lung) OR (pulmonary)) AND ((neonatal) OR (pediatric)) reveals that the number of articles published matching these criteria has increased dramatically over the last 20 years, with a ~10‐fold increase between 2000 and 2021. Alongside this, novel treatments for several lung diseases that have made a significant impact on lung health are becoming more readily available to patients, and there is a need for safe methods for longitudinal monitoring of lung structure and function to accompany these often‐expensive treatments. In particular, the advent of triple combination therapy for CF has transformed quality of life in those who are genetically eligible for it,^6^ and biologics show dramatic suppression of asthma exacerbations.^7^ Conventional MRI is very safe in children and can be repeated throughout childhood as a monitoring tool.^8^ The Fleischner society recently published a position paper on the clinical status of lung MRI, recommending clinical use of ^1^H MRI in children with CF^9^ and the European Respiratory Society (ERS) highlighted the potential importance of free‐breathing ^1^H lung MRI methods in bronchopulmonary dysplasia (BPD) assessment.^10^

There are several challenges in obtaining high quality MR images of the lung structure or function in children. The low proton density (20% ~ 40% of that of chest wall muscle^11^) and air‐tissue susceptibility gradient driven short T_2_* (~2 msec at 1.5 T, <1 msec at 3 T^12^) generally constrain ^1^H lung MRI, though a recent resurgence in diagnostic quality low‐field MR (T_2_* lung ~10 msec at 0.55 T^13^) is poised to overcome the latter. Dealing with motion—not only respiratory and cardiac, but also bulk motion of the subject, is of paramount importance in children. In adults, the normal range of respiratory rate is around 12–20 breaths per minute. Respiratory rates are higher in children and particularly rapid in infants; the median respiratory rate is approximately 44 breaths per minute at birth (up to 1 Hz in severe disease^14^) and 26 breaths per minute at 2 years old.^15^ Rapid imaging during short breath‐holds for compliant patients, or during free‐breathing with respiratory gating and/or retrospective signal processing are critical, as are pre‐exam communication with the patient, and techniques to minimize distress in the scanner—such as low acoustic noise sequences, A/V equipment for distraction etc.—to allow the exam to proceed without the use of sedation.

This review is structured in two parts:

- an overview of the various MRI methods that have found utility in pediatric and neonatal lung MRI, with reference to the aspects of lung structure and function that they can probe, and how motion is dealt with,
- highlight of recent applications of these methods in children and neonates with lung disease, with a particular focus on the most‐widely studied diseases to date, namely BPD and CF.

While other relatively recent reviews have been published focusing on either school‐aged children or neonates.^16^, ^17^ However, there is a need for a comprehensive analysis of neonates, pre‐school, and school‐aged children to address the similarities and differences in MRI studies of these populations.

## Methods for Pulmonary MRI in Newborns and Children

### T_1_ ‐, T_2_ ‐Weighted

**FIGURE 1:**
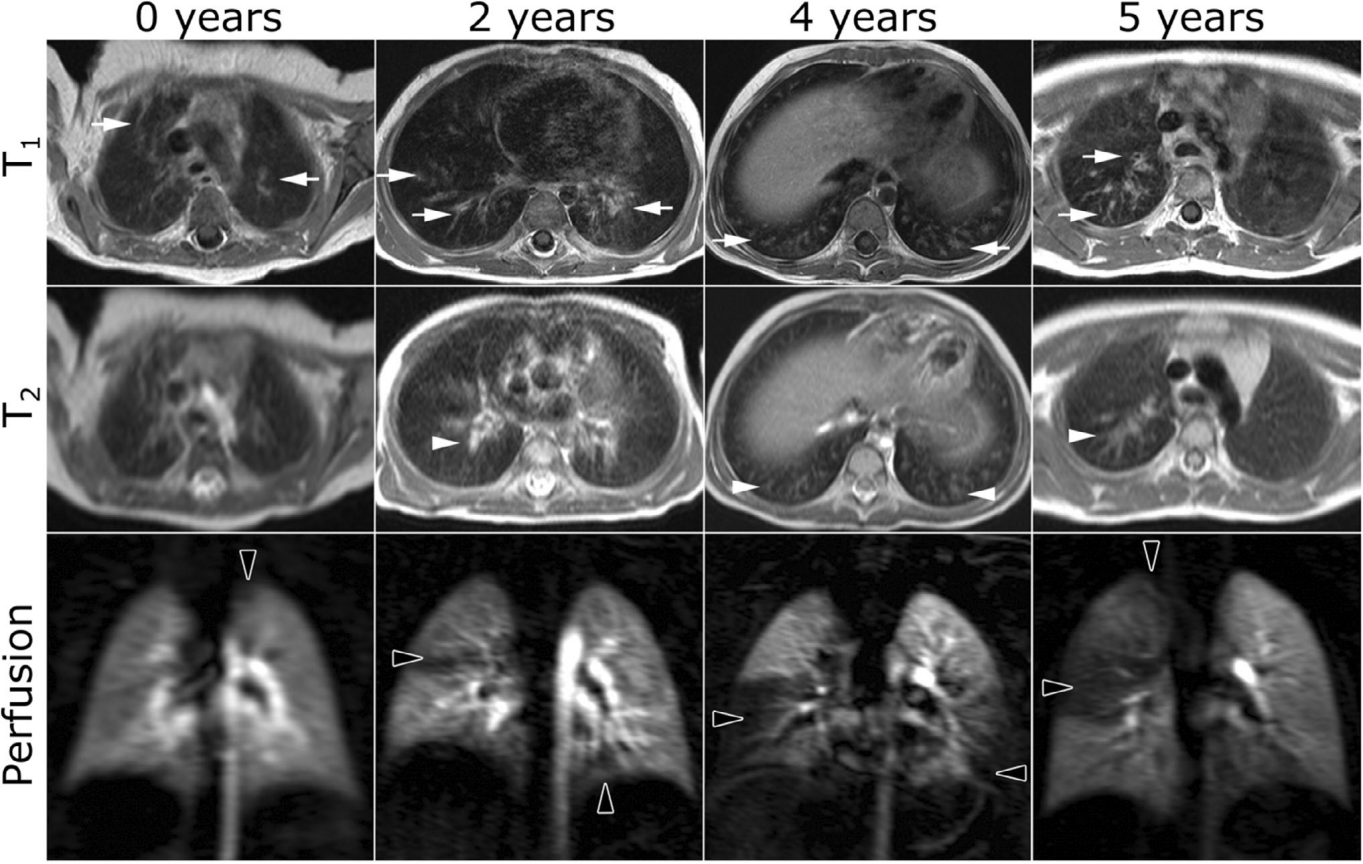
Structural T_1_‐weighted gradient echo and T_2_‐weighted (HASTE) imaging alongside gadolinium contrast enhanced perfusion imaging in infants and children 0–5 years old with cystic fibrosis. Reprinted with permission of the American Thoracic Society.^1^ Copyright © 2024 American Thoracic Society. All rights reserved. The American Journal of Respiratory and Critical Care Medicine is an official journal of the American Thoracic Society. White arrows: airway structural abnormalities including wall thickening and/or bronchiectasis, white arrowheads: mucus plugging, black arrowheads: perfusion abnormalities. HASTE = half‐Fourier single‐shot turbo spin echo.

Fast gradient echo and spin echo sequences are common constituents of pediatric chest MRI protocols,^18^ due to their simplicity of implementation and feasibility to be acquired within short breath‐holds suitable for children. 3D T_1_‐weighted gradient echo sequences are commonly used for lung volumetry,^19^ and are feasible for ungated morphological imaging in infants.^20^ In addition, such sequences can be performed dynamically to evaluate airway dynamics, eg, tracheobronchomalacia in children.^21^ Rapid, balanced steady‐state free precession (bSSFP) sequences with their mixed T_1_–T_2_ contrast are a widely used in cardiovascular imaging, and in the lungs provide strong contrast between blood vessels and lung parenchyma.^22^, ^23^ Despite their relatively long echo times with respect to the short T_2_* in the lung, T_1_ and proton‐density weighted spin echo sequences can also provide information about pathological water content, eg, as demonstrated in the lungs of preterm infants.^24^, ^25^ Fast spin echo approaches, in particular half‐Fourier single‐shot turbo spin echo (HASTE), provide good contrast of the parenchyma^26^ and are suitable for breath‐hold morphological assessment^27^ and detection of infiltrates.^28^ While T_2_‐weighted spin‐echo sequences are generally sensitive to “plus pathologies” such as inflammation, fibrosis, atelectasis, mucus plugging, short‐TE gradient echo sequences with T_1_ or proton density contrast can provide sensitivity to “minus pathologies,” eg, alveolar simplification, cysts, air trapping. Example T_1_‐ and T_2_‐weighted images obtained from infants and children with CF (0–5 years) are shown in Fig. 1. The increased robustness to motion provided by non‐Cartesian acquisition methods such as Periodically Rotated Overlapping ParallEL Lines with Enhanced Reconstruction (PROPELLER) makes them attractive for applications in infants and non‐compliant children. Typically T_2_‐weighted, PROPELLER offers comparable contrast to HASTE sequences, and can be tailored to increase proton‐density contrast; this has been utilized to assess CF lung disease in children and adolescents though with lower sensitivity than CT.^29^

### Contrast‐Enhanced Imaging

Dynamic contrast‐enhanced (DCE) MRI is a straightforward and easy‐to‐implement technique for imaging lung perfusion. Contrast agents are typically gadolinium‐based with a safety profile that has been well studied in infants and children.^30^ Rapid 3D gradient echo based T_1_‐weighted acquisitions—with parallel imaging, segmented k‐space acquisition with view sharing (eg, Reference 31) and/or compressed sensing^32^ for maximal temporal resolution—allow the visualization of an intravenously injected contrast bolus as it circulates through the pulmonary arterial system, the left heart, the aorta, and the bronchial circulation. DCE perfusion acquisitions are included within pediatric protocols used clinically in some centers for assessment of CF^33^ and congenital lung malformations,^34^ though adoption of DCE methods in children is not widespread at present. Assessment of the necessity of DCE MRI in pediatric lung disease in light of evidence of gadolinium deposition^35^ is likely to vary by center/country and the clinical question at hand. Example images acquired from infants and children with CF (0–5 years) are shown in Fig. 1.

### Non‐Cartesian Acquisitions & Ultra‐Short/Zero Echo Time

**FIGURE 2:**
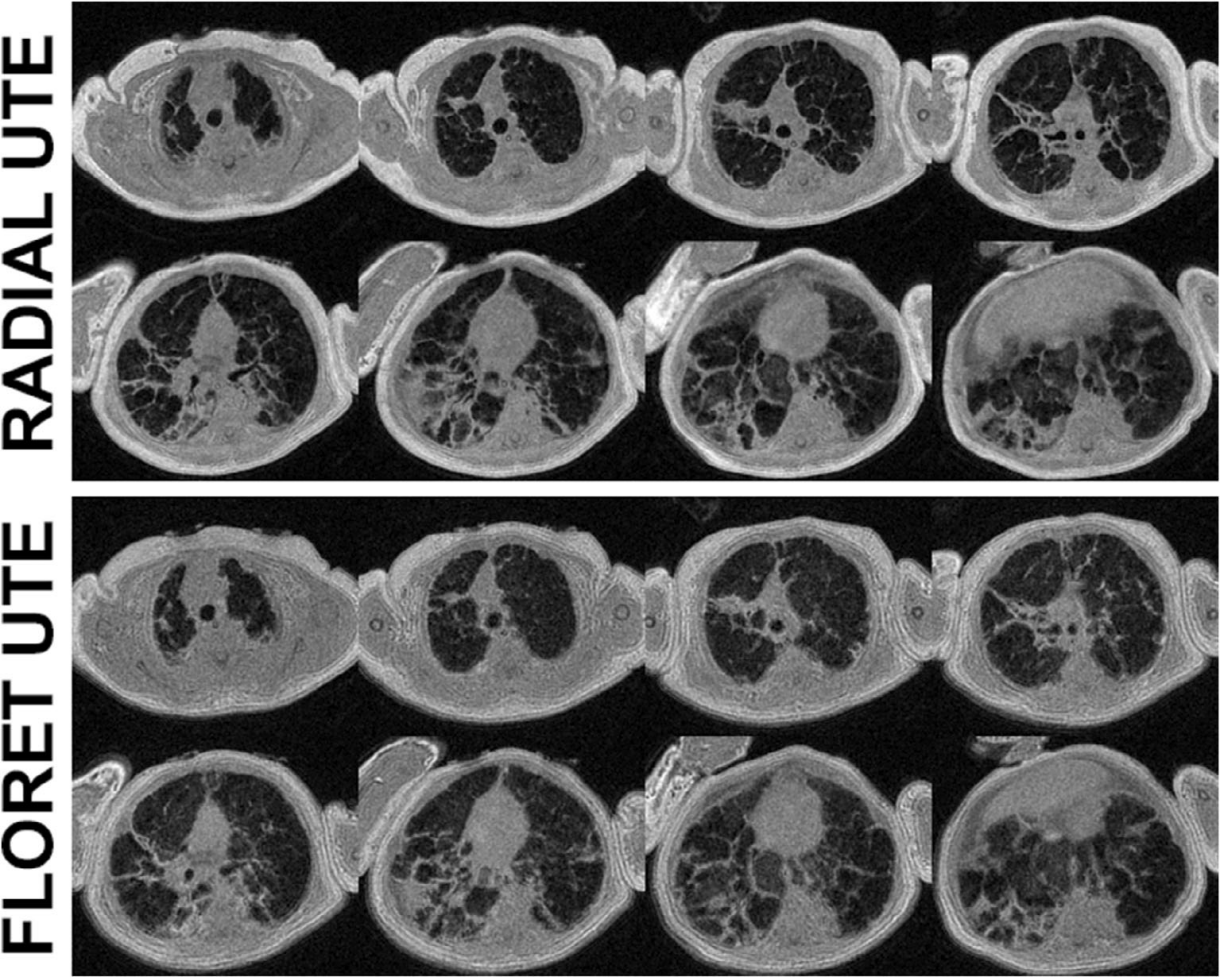
Comparison of UTE structural lung images acquired with 3D radial and FLORET trajectories in a neonate with bronchopulmonary dysplasia. Both images were acquired with 180 mm^3^ FOV and 0.7 mm isotropic resolution. Scan time was 16 minutes 44 sec for 3D radial UTE and 4 minutes 41 sec for FLORET UTE. (Images provided courtesy of Professor Sean Fain and Dr Andrew Hahn at University of Iowa.) FLORET = fermat‐looped, orthogonally encoded trajectories; FOV = field of view; UTE = ultrashort echo time.

Non‐Cartesian (radial, spiral etc.) acquisitions with center‐out k‐space read‐outs can counteract the rapid T_2_* decay of proton signal in the lung tissue, providing improved signal‐to‐noise. An optimized 3D radial UTE acquisition with variable‐density read‐out and coarse 3D slab selection has been developed^36^ and implemented in a number of sites for adult and pediatric lung imaging, providing improved proton‐density weighting with preserved signal in lung parenchyma compared with conventional gradient echo methods. The long scan times of 3D radial UTE approaches necessitate acquisition during quiescent breathing in combination with some form of respiratory gating (prospective or retrospective), and with a pseudorandomized view order to allow for reconstruction with an arbitrary subset of acquired data. Respiratory bellows can be used in older children but present challenges in infants^14^; center‐out acquisitions inherently repeatedly sample *k*_0_, which provides an alternative “self‐navigated” motion tracking waveform, allowing for retrospective discarding of data corrupted by bulk motion and reconstruction of images with reduced motion artifact. The 3D radial approach has been adapted for neonates to provide high spatial resolution (~0.7 mm^3^) and full chest coverage in non‐sedated, tidal‐breathing infants with and without lung disease,^37^ and in a follow‐up study, the proton‐density‐weighted UTE lung intensity was found to be regionally associated with CT lung density in infants with a wide range of normal and pathological lung densities.^38^ While 3D radial performance is generally high in infants and children, the long acquisition times (~5 to 15 minutes depending on FOV) are not ideal for rapid clinical throughput, and alternatives have been explored. Pseudo‐3D stack‐of‐spirals methods have been used (predominantly on Siemens systems) to greatly reduce the acquisition time at the expensive of susceptibility to motion artifacts^39^; such approaches may even be feasible at breath‐hold for resolutions ≥2 mm^3^ in compliant children.^40^ In addition, Fermat‐looped, orthogonally encoded trajectories (FLORET)^41^ (primarily developed on Philips systems), can offer significantly reduced scan times (~25%–40% that of 3D radial), while preserving image quality, resolution, and retrospective gating capabilities. The feasibility of FLORET has been investigated in pediatric subjects with CF,^42^ and is under ongoing study in pre‐term infants (Fig. 2).^43^

Zero echo time (ZTE) methods—in which the spatial encoding gradients are switched on at the time of the radiofrequency pulse excitation—show promise for structural lung imaging,^44^ having similar contrast to UTE, and can be acquired during free‐breathing with retrospective gating to investigate lung function.^45^ The low acoustic noise footprint of these sequences is attractive for pediatric and especially neonatal applications to improve compliance and patient comfort, for example reducing the chance of subjects awakening when imaging during asleep, however such sequences are yet to show the same diagnostic performance as UTE.^46^

**FIGURE 3:**
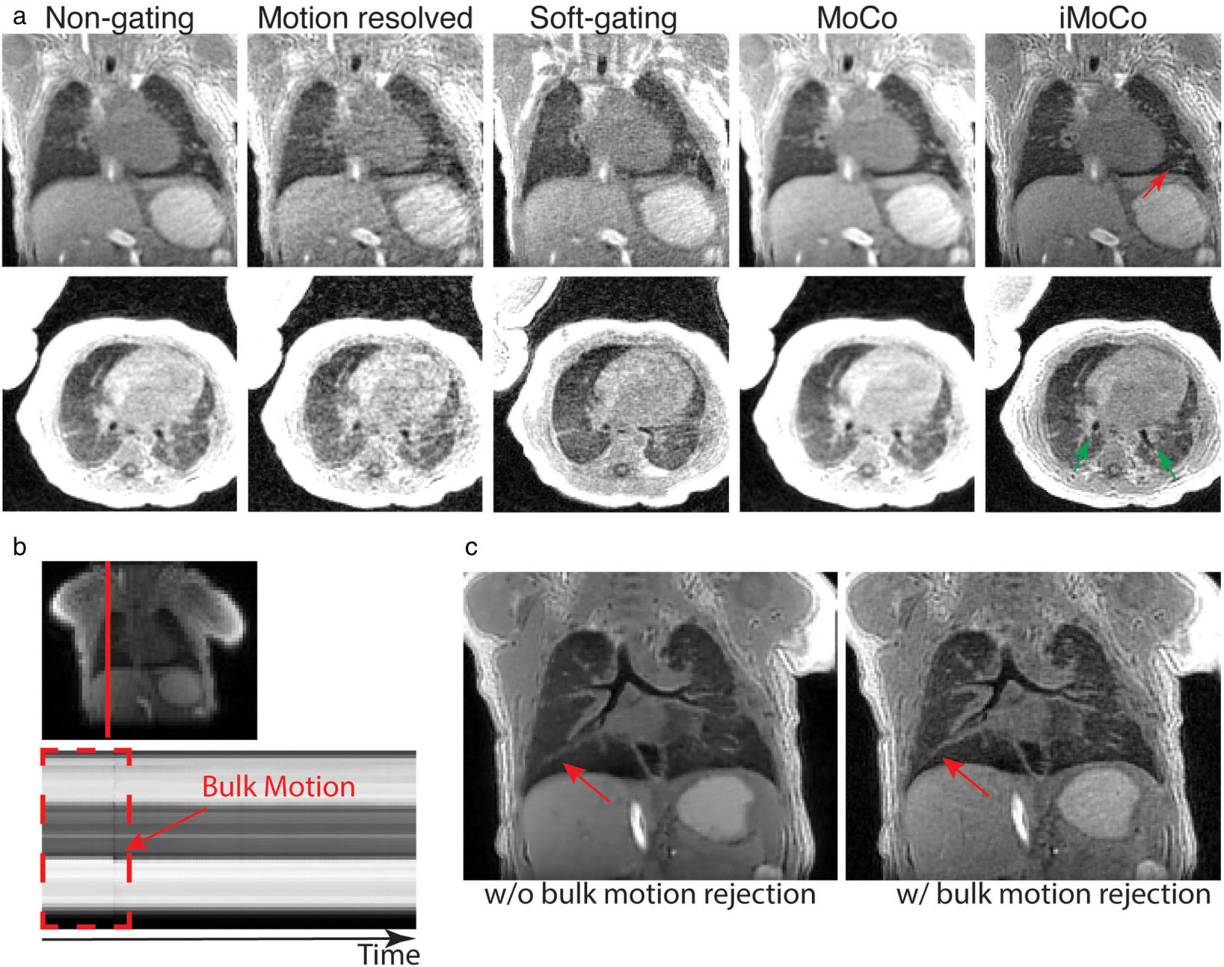
(**a**) Illustration of improved motion compensation and signal‐to‐noise ratio of iterative motion compensation (iMoCo) reconstruction compared with non‐gated, XD‐GRASP‐like motion‐resolved, soft‐gated reconstructions, and non‐iterative motion compensated (MoCo) reconstructions in a 10‐week‐old infant. Reprinted with permission from Reference 49. Red arrows indicate vessels and green arrows the airways visualized on the iMoCo images. (**b**) Image‐based motion navigator, iMoCo images without (**c**) and with (**d**) bulk motion rejection showed reduced motion blurring after bulk motion compensation. (Data highlighted by the red‐dash rectangle were removed prior to reconstruction as they were acquired prior to a bulk movement; red arrow.) XD‐GRASP = extra‐dimensional golden‐angle radial sparse parallel.

Beyond retrospective respiratory gating to reconstruct images at end expiration, continuous acquisition of 3D UTE data throughout the breathing cycle allows for reconstruction at various inflation states. Tidal volumes can be calculated from images reconstructed at end expiration and inspiration, and agreement between such UTE‐measured tidal volumes and those predicted by physiologic scaling has been reported in neonates.^14^ Furthermore, after registering images to a reference respiratory state, signal intensity differences between respiratory state images can be compared to calculate the fractional ventilation—a measure of ventilation akin to that measured by hyperpolarized gas MRI and PREFUL as discussed in the following subsections, and a subject of ongoing research.^39^, ^47^ In recent years, a wealth of motion‐compensated/motion‐resolved image reconstruction techniques have been proposed to improve the quality of retrospectively gated reconstructions. XD‐GRASP incorporates respiratory motion as an additional regularization term into an iterative compressed sensing reconstruction,^48^ while Zhu et al proposed an iterative motion compensation (iMoCo) strategy that instead incorporates motion fields from image registration of respiratory‐motion‐resolved UTE images into the CS algorithm.^49^ iMoCo UTE images in pediatric and infant patients (Fig. 3) demonstrated clear pulmonary structures, and both higher apparent SNR and CNR compared to other motion correction strategies. A challenge of the multi‐step iMoCo strategy is its dependence on image quality at each post‐processing step. To address this, Zou et al introduced motion‐compensated smoothness regularization on manifolds (MoCo‐SToRM),^50^ an unsupervised deep‐learning scheme for MoCo reconstruction for application in free‐breathing lung MRI. This study experimentally demonstrated improved structural resolution with MoCo‐SToRM compared with iMoCo and XD‐GRASP in four adults and one infant with BPD, with improvement particularly pronounced when a scan was corrupted by multiple bulk motion events. Most recently, motion‐compensated low‐rank reconstruction (MoCoLoR) has been developed to add a further low‐rank constraint on the motion fields, and has ventilation mapping with both signal intensity based and deformation field‐based methods has been reported.^51^

A technique combining highly undersampled 2D radial or 3D stack‐of‐stars methods with rapid, serial image reconstruction—dubbed “real‐time MRI”^52^—has recently shown promise for volumetric coverage of the chest in children on a time scale of the order of tens of seconds with high robustness to respiratory motion.^53^ This approach is currently limited by the requirement for a custom reconstruction set‐up with dedicated multi‐GPU machine for real‐time reconstruction, but if demonstrated to be scalable, could help dramatically reduce the duration of high‐resolution scans for morphological lung MRI exams.

### Inhaled Hyperpolarized Gas Imaging

Hyperpolarized gas MRI has matured into an important modality for imaging the function and microstructure of the lung.^4^ While several of the landmark studies referenced herein have been performed using ^3^He gas, its heavily restricted availability and high cost have moved the field toward ^129^Xe gas over the last 5 to 10 years; moreover, ^129^Xe offers additional functionality with its ability to probe gas exchange function. The key aspects of lung function accessible with hyperpolarized gas MRI and the methods used in each case are described briefly here. For further details, we refer the reader to protocol reference paper published by the ^129^Xe MRI Clinical Trials Consortium (note: these protocols are recommended for imaging of adult subjects).^54^

#### VENTILATION

2D/3D gradient echo imaging during a short breath‐hold after inhalation of a dose of hyperpolarized gas can be used to visualize the gas delivery to the lungs and its distribution, i.e., the lung ventilation and its heterogeneity. Beyond qualitative assessment, ventilation abnormality is most‐commonly quantified by the ventilation defect percentage (VDP); the percentage of lung that yields no signal, i.e., is not ventilated. Ventilation heterogeneity can further be quantified by histogram analysis and the coefficient of variation of ventilated signal^55^ and images can be presented as maps colored by binning the signal according to a healthy reference distribution.^56^ In addition, imaging can be performed repeatedly to study ventilation *dynamics* and derive maps of the gas turnover per breath known as fractional ventilation.^57^ This has been shown to be feasible in pediatric participants^58^ and provide regional information comparable to the global information obtained from the multi‐breath washout pulmonary function test—a sensitive marker of ventilation heterogeneity.

#### MICROSTRUCTURE

Diffusion‐weighted imaging of hyperpolarized gases is feasible by inserting bi‐polar diffusion gradients of modest strength into a spoiled gradient echo sequence. The apparent diffusion coefficient (ADC) of hyperpolarized gases in the lungs is reduced due to restriction by the alveolar walls; a two *b*‐value measurement of ADC therefore provides a simple metric of alveolar size that is highly sensitive to, eg, emphysematous tissue destruction.^59^ Diffusion in the lungs is known to be non‐mono‐exponential in nature, and multiple (>2) *b*‐value diffusion‐weighted imaging of hyperpolarized gases has been explored to better model the signal behavior and relate it to microstructural parameters. Notably, the cylinder model which treats the airways as cylinders and introduces two orthogonal diffusion coefficients,^60^ and the stretched exponential model which introduces a diffusion heterogeneity index.^61^ The application of these models is compared in patients with obstructive and restrictive lung diseases in Chan et al.^62^

#### GAS EXCHANGE

Upon inhalation, xenon partially dissolves in the lung parenchyma and pulmonary capillaries; ^129^Xe exhibits discrete chemical shifts in the parenchyma and blood plasma (collectively “membrane”) and the red blood cells, approximately 200 ppm downfield from the resonance of ^129^Xe gas in the alveoli. Information about gas exchange—a key function of the lung—can be obtained from the ^129^Xe signals in these different environments by ^129^Xe MR spectroscopy.^63^ Time‐resolved spectroscopy and modeling of ^129^Xe uptake in the membrane and red blood cells using chemical shift saturation recovery can provide quantitative measures of lung microstructure and function.^64^, ^65^ Spatial localization can be achieved through chemical shift imaging techniques, typically 3D UTE radial in design to overcome the short 1–2 msec T_2_* of dissolved ^129^Xe, with chemical shift separation according to the 1‐point Dixon, whereby the echo time is chosen to induce a 90° phase difference between M and RBC resonances,^66^ or using multi‐echo approaches with matrix inversion^67^ or IDEAL decomposition.^68^

Quantitative assessment of pathological changes in hyperpolarized gas MRI metrics often involves comparison with a reference age‐matched healthy cohort. Recently, efforts have been made to establish such reference distributions for ^129^Xe MRI gas exchange metrics in healthy children,^69^ and study their variation with age.^70^ In the future, the use of predicted metric values may be envisaged in the same manner as that of pulmonary function tests.^71^

The doses used for ^129^Xe MRI—typically ≤1 L—and short breath‐hold procedure are safe and well below the threshold required to induce anesthesia.^72^ Walkup et al reported 100% tolerability and no serious or severe adverse events associated with xenon inhalation in children aged 6–16 years (including healthy control subjects and participants with CF).^73^ As is observed in adults, a decrease in SpO_2_ ~6% was observed in most children, and some children experienced mild side effects such as dizziness, euphoria; all of which resolved quickly after the inhalation.^73^ There is little safety data in children under the age of 5–6 years old, aside from a report on the feasibility of ^129^Xe MRI in premature newborns with BPD, wherein a similar transient SpO_2_ decrease of ~4.5% was observed.^74^

It is worth noting that in recent years, perfluorinated gases have shown some promise as inhaled MR contrast agents for imaging lung ventilation and microstructure.^75^ While not as sensitive and technologically mature as ^129^Xe lung imaging at present, ^19^F lung MRI has several advantages; no hyperpolarization requirement, short T_1_ and can be imaged rapidly over multiple breaths and averaged to provide reasonable SNR,^76^ similar gyromagnetic ratio to ^1^H reducing the need for specialized RF coils. A preliminary report suggests this method may be feasible in children.^77^

### Non‐Contrast ^1^H MRI Ventilation & Perfusion: Fourier Decomposition, PREFUL & OE‐MRI

**FIGURE 4:**
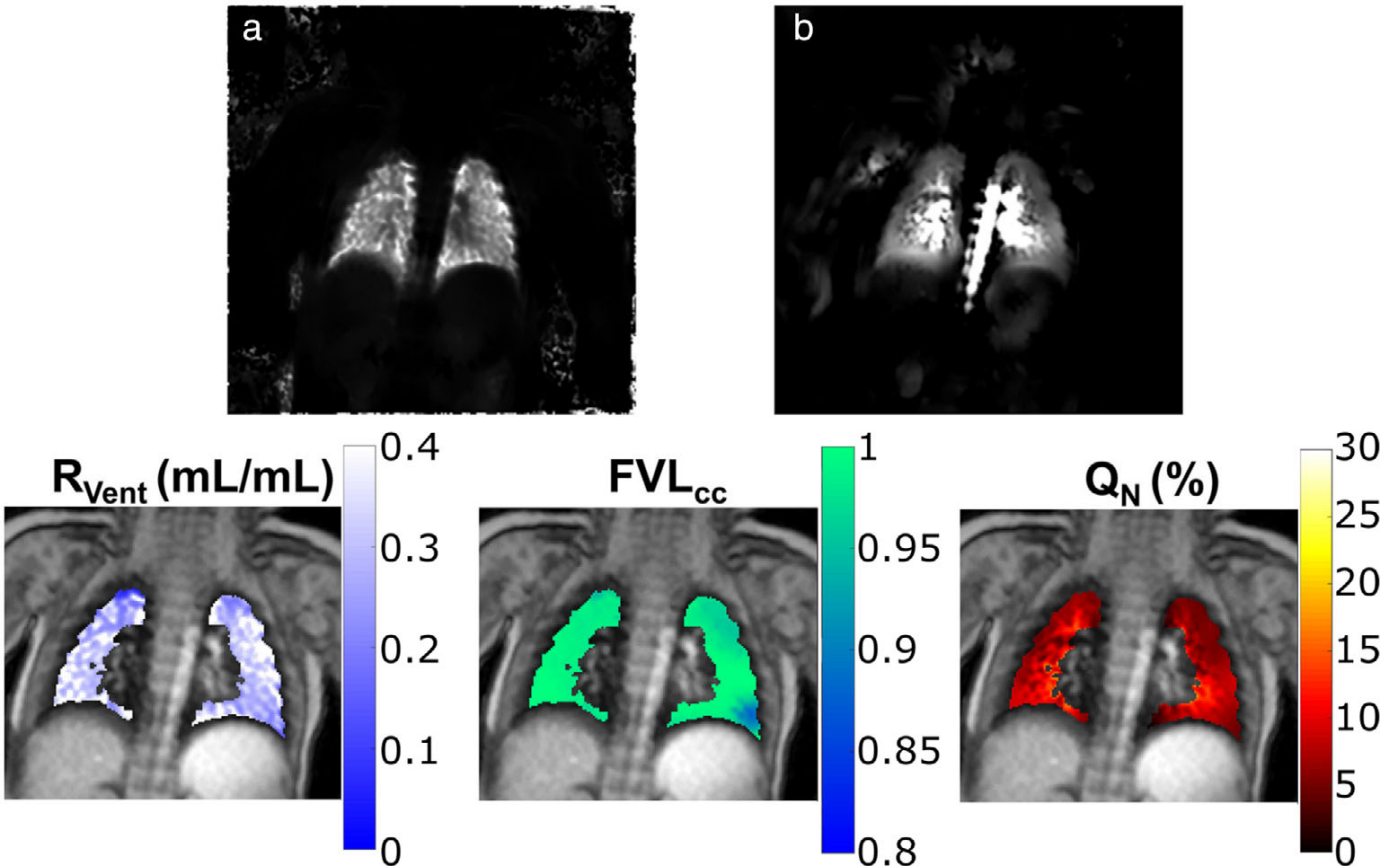
PREFUL MRI results obtained from an 8‐month‐old infant using a 2D gradient echo sequence. Reprinted with permission from Reference 82. (**a**) Ventilation‐, (**b**) perfusion‐weighted maps. *R*_vent_ = regional ventilation; FVL_cc_ = flow‐volume loop cross correlation; *Q*_*N*_ = normalized perfusion percentage maps; PREFUL = phase‐resolved functional lung.

Rapid (>3 frames/sec), repeated acquisition of balanced/spoiled gradient echo images during free‐breathing, subject to spatial co‐registration, yields a series of images with temporal changes in parenchymal signal that are dependent on the position in the respiratory and cardiac cycles. Fourier decomposition is a means to separate this signal‐time behavior according to the respiratory and cardiac frequencies and produce maps of ventilation and perfusion, respectively.^78^ Over the last 10 years, several developments and variations on the original FD method have been developed. Notably, SElf‐gated Non‐Contrast‐Enhanced Functional Lung imaging (SENCEFUL)^79^ adds the acquisition of a *k*_0_/DC navigator to provide the respiratory/cardiac modulated signal, and matrix pencil (MP) decomposition has been proposed to improve the robustness of FD MRI for estimating respiratory and cardiac frequencies.^80^ Most recently, phase‐resolved functional lung (PREFUL) has been developed as an alternative approach to process the registered time‐series of images to obtain full respiratory and cardiac cycle reconstruction and subsequent phase‐based analyses.^81^ Zanette et al have demonstrated the feasibility of the PREFUL approach to map regional ventilation and perfusion in healthy infants (Fig. 4).^82^ While typically 2D methods are used for FD methods due to the high temporal resolution requirement, PREFUL has been recently extended to 3D using a stack‐of‐stars approach.^83^

Oxygen‐enhanced MRI, which uses the T_1_‐shortening property of paramagnetic molecular oxygen presents an alternative, widely available contrast agent for assessment of pulmonary ventilation.^84^ Aside from one successful demonstration of T_1_ mapping under different hyperoxic conditions in a study that included adolescents and young adults with CF,^85^ this technique has had limited application in children. Clinical uptake has been further constrained by the relatively modest signal enhancement and the complication of multiple contributing physiological factors to the signal enhancement.

Take‐home messages/Recommendations:

- Conventional T_1_‐ and T_2_‐weighted sequences provide complementary contrast in the lungs to locally increased air, and fluid, respectively, characteristic of pathological features
- 3D UTE imaging is a non‐ionizing alternative to CT
- DCE MRI is only used in a few centers in pediatrics, but substantial safety data is available
- PREFUL is the most widely applied of existing post‐processing methods for mapping ventilation and perfusion from free‐breathing ^1^H MRI
- Hyperpolarized ^129^Xe is a direct, sensitive measure of lung ventilation, and recent efforts to standardize ^129^Xe MRI metrics of gas exchange and their age‐dependence should increase its utility in children
- Several recommended protocols have been published (eg, References 86, 87), though most only include conventional ^1^H sequences. Niedbalski et al published a recommended protocol for hyperpolarized ^129^Xe lung imaging in adults.^54^ We provide a table summarizing these approaches and when they should be used, alongside references for implementation (Table 1). For GE Healthcare scanners we provide a protocol on the web that can be filtered for pediatric cases (https://polaris-sheffield.github.io/sheffield-lung-protocol/).

**TABLE 1:** Suggested Pulse Sequences to Comprise a Pediatric Lung MRI Protocol

Sequence	Plane	Contrast	Indication	Usage/Comment	Breath‐Hold*	Ref
^*1*^ *H Structural*				*Core components*		
3D spoiled GRE^a^	Coronal	T_1_/PD	Lung volume, air trapping	Optionally can be performed at RV to identify air trapping, and TLC	<10 s	18, 115
2D bSSFP^b^	Axial	T_1_ & T_2_	Blood vessels, mucus, etc.	Optionally performed in free breathing where 50% slice overlap is recommended	<10 s or free‐breathing	22, 86
3D UTE/ZTE	Axial/Isotropic	PD	Alveolar simplification, cysts, air trapping, etc.	Product sequences not yet available for all vendors	Free‐breathing (w/bellows/self navigation)	14, 36
2D SSFSE^c^	Axial/Coronal	T_2_	Inflammation, mucus plugging, etc.	Can be interchanged with PROPELLER	<10 s	18, 27
PROPELLER^d^	Axial	T_2_	Inflammation, mucus plugging, etc.	Can be interchanged with 2D SSFSE	Free‐breathing (w/navigator)	29, 108

^*1*^ *H Functional*				*Optional/Local Preference*		
2D/3D Fourier Decomposition	Coronal		Ventilation & perfusion (2D only)	Spoiled/balanced GRE. Post‐proc available on‐line for Siemens only	Free‐breathing	140, 168
3D dynamic SPGR with contrast^e^	Coronal	T_1_ (w/contrast)	Perfusion	Recommended only for clinical queries re: perfusion deficit	≤20 s breath‐hold in older children or Free‐breathing (w/bellows/self navigation)	33

^129^ *Xe Functional*				*Requires* ^*129*^ *Xe polariser + multi‐nuclear capable scanner*		
2D SPGR/3D bSSFP	Coronal		Ventilation	2D Cartesian implementation used in multi‐site trials	<10 s	54, 169
2D/3D DW SPGR	Coronal/Axial		Alveolar airspace size	Recommended only for certain conditions (eg, BPD)	Ideally <10 s but not yet widely achieved	105, 170
3D radial Dixon or multi‐echo	Isotropic		Gas exchange	Recommended only for certain conditions (eg, ChILD, BPD)	Ideally <10 s but not yet widely achieved	70

^a^ FLASH or VIBE (Siemens), SPGR (GE Healthcare), FFE (Philips).

^b^ TrueFISP (Siemens), FIESTA (GE Healthcare), Balanced FFE (Philips).

^c^ HASTE (Siemens), SSFSE (GE Healthcare), SSTSE (Philips).

^d^ BLADE (Siemens), PROPELLER (GE Healthcare), MultiVane (Philips).

^e^ With view‐sharing, eg, TWIST or TWIST‐VIBE (Siemens), TRICKS or DISCO (GE Healthcare), 4D‐TRAK.

* Breath‐holds are recommended for children aged 5 years and above.

## Applications of Pulmonary MRI in Newborns and Children

### Lung Disease in Infants: Bronchopulmonary Dysplasia & Congenital Diaphragmatic Hernia

In recent years, pulmonary MRI has found clinical and pathological relevance in infants, particularly in those born prematurely who have developed BPD, and those with congenital disorders that affect pulmonary development (eg, congenital diaphragmatic hernia [CDH], pulmonary hypoplasia).

**FIGURE 5:**
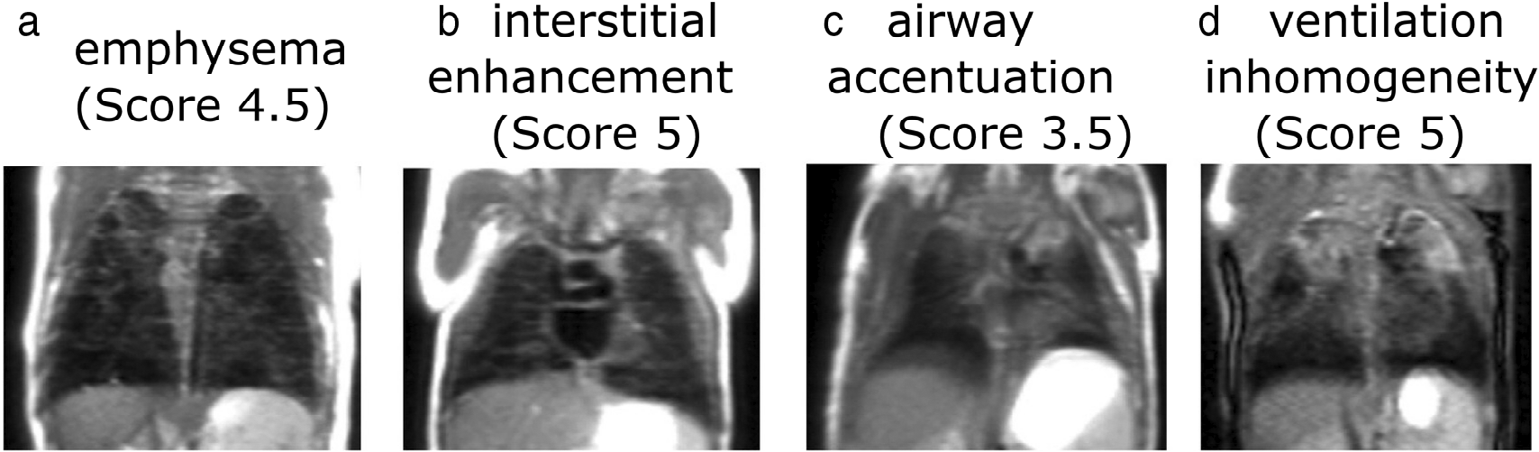
Examples of HASTE images showing the key features of the UNiforme Scoring of the disEAsed Lung in bronchopulmonary dysplasia (UNSEAL BPD) scoring system. Reprinted with permission from Reference 91. Each feature is scored from 1 to 5 where 1 is absence and 5 is global high prevalence/severe disease. HASTE = half‐Fourier single‐shot turbo spin echo.

The most mature evaluations of lung structure in neonates have used reader‐based scores. Higano et al implemented a scoring system modified from the Ochiai lung CT score^88^ to assess high‐resolution UTE images of lung structures in neonatal intensive care unit (NICU) inpatients with BPD.^89^ This system evaluates pulmonary abnormalities including hyperexpansion, emphysema, fibrotic/linear opacities, mosaicism, bronchovascular bundle distortion, and subjective impression^20^ (Table 2). In a cohort of 42 infants, MRI scores correlated significantly with BPD severity gradings, and furthermore, in multivariable modeling that also included several clinical parameters, MRI lung scores were the most significant predictor of the duration of any respiratory support, positive pressure support, and invasive ventilator support. This scoring scheme was then used with relevant clinical variables to create a binomial logistic regression to predict BPD patients' early risk of tracheostomy requirement^90^; the combined model performed better than models that only used MRI scores or clinical data. Förster et al devised a bespoke scoring system for T_2_‐weighted single‐shot fast spin echo lung images (UNiforme Scoring of the disEAsed Lung in BPD, UNSEAL BPD^91^) (Fig. 5) that characterizes abnormalities including interstitial and airway remodeling, emphysematous changes, and ventilation inhomogeneity, with more emphasis on water content (eg, consolidation, bronchial wall edema, and mucus plugs) than with the modified Ochiai system for proton density‐weighted images. The UNSEAL BPD score was able to identify infants with moderate and severe BPD disease grades, and structural changes present at term‐age imaging that were specific to patients' gestational age at birth; the most premature infants presented with increased emphysema scores, while infants born after 26 weeks gestation had increased scores for interstitial enhancement, suggestive of fibroproliferative remodeling.

**TABLE 2:** Modified Ochiai Scoring Scheme for Features of Bronchopulmonary Dysplasia on Structural Lung MRI

Feature	Scoring		
	0	1	2
1. Hyperexpansion	None	Focal	Global
2. Mosaic lung attenuation	None	Unclear	Obvious
3. Emphysema, number of cysts/regions	None	Single	Multiple
4. Emphysema, size	None	<5 mm	>5 mm
5. Fibrous/interstitial, triangular subpleural opacities	None	1–3 lobes	4–6 lobes
6. Distortion of bronchovascular bundles	Mild	Moderate	Severe
7. Subjective impression	Mild	Moderate	Severe
*Maximal score*	14		

Scoring scheme taken from References 20, 89. (See Reference 88 for example images depicting these pathological features.)

Beyond subjective reader scoring, more quantitative approaches are emerging to assess abnormal lung structures in infants, such as parametric mapping. Higano et al demonstrated proton‐density mapping in infants with and without BPD and quantified the percentage of whole‐lung tissue at abnormally high and low densities^92^; this percentage strongly correlated with clinical BPD severity levels and with relevant subscores from the modified Ochiai scoring system. Multi‐echo UTE has been used to map R_2_* as a novel measure of lung microstructure; in control infants a baseline was established for lung parenchymal R_2_* at near term age of 335 ± 65 s^−1^, which was significantly lower than in healthy adults (465 ± 15 s^−1^).^93^ In a follow‐up study, the R_2_* in infants with BPD and ipsilateral CDH were found to be non‐significantly higher than control infants, and R_2_* in contralateral CDH was significantly higher.^94^ The inverse relationship between tissue proton density and R_2_* was influenced by the presence of disease, though with as‐yet undetermined links to structural or physiological mechanisms.

Straightforward anatomical metrics have played an early but important role in understanding the asymmetric structural development and potential for pulmonary catch‐up growth in infants with pulmonary hypoplasia, particularly CDH. Schopper et al measured prenatal and postnatal lung volumes using FSE and SPGR sequences in the ipsilateral and contralateral lungs of infants with left‐sided CDH to investigate potential compensatory growth,^95^ and found that lung volume grew faster in severe CDH compared with mild CDH, and that total lung volume growth in moderate CDH was predominantly attributable to growth of the ipsilateral lung. To investigate whether this growth represented true parenchymal development or simply expansion of existing airspaces, Adaikalam et al used 3D UTE to measure normalized lung signal intensities and lung volumes, and calculated ipsilateral and contralateral lung mass in patients with CDH.^96^ When compared to lung mass calculated from fetal MRI, the ipsilateral mass growth rate was higher than the contralateral rate, evidencing “catch‐up” growth of lung mass in the more hypoplastic ipsilateral lung. Advancements in AI‐based segmentation have made 3D lung volumetry a relatively trivial task; Mairhörmann et al recently reported that lung volumes and associated structural feature parameters derived from automated segmentations of HASTE images in premature babies were sensitive to BPD grade.^97^ In particular, the lung elongation feature was found to be particularly complementary to conventional radiomic features.

Most approaches for assessing neonatal lung function rely on dynamic acquisition and reconstruction strategies that capture many lung inflation states. Using self‐navigating 3D radial UTE in infants with severe BPD, Gouwens et al demonstrated that tidal volumes in cystic lung regions were higher than non‐cystic regions, and that clinical ventilator settings for peak inspiratory pressure did not correlate with cystic tidal volumes.^98^ Similar methods were used to generate end‐expiratory and end‐inspiratory gated UTE images in infants with BPD, showing that total lung volumes at functional residual capacity (FRC), tidal volumes, and minute ventilation were correlated with increasing severity grade.^99^ Large total lung volumes (i.e., overinflation) were considered an imaging biomarker for small airways obstruction and air trapping. In a multi‐center study, Katz et al used a breath‐hold UTE sequence at expiration and inspiration to derive proton density measures that were sensitive to BPD grade in school‐aged children born extremely pre‐term.^100^ Using PREFUL strategies as per,^82^ Dyke et al showed regional variations in ventilatory function in preterm NICU inpatients and a significant difference in total VDP between groups of no BPD, BPD grade 0/1, or BPD grade 2/3.^101^

**FIGURE 6:**
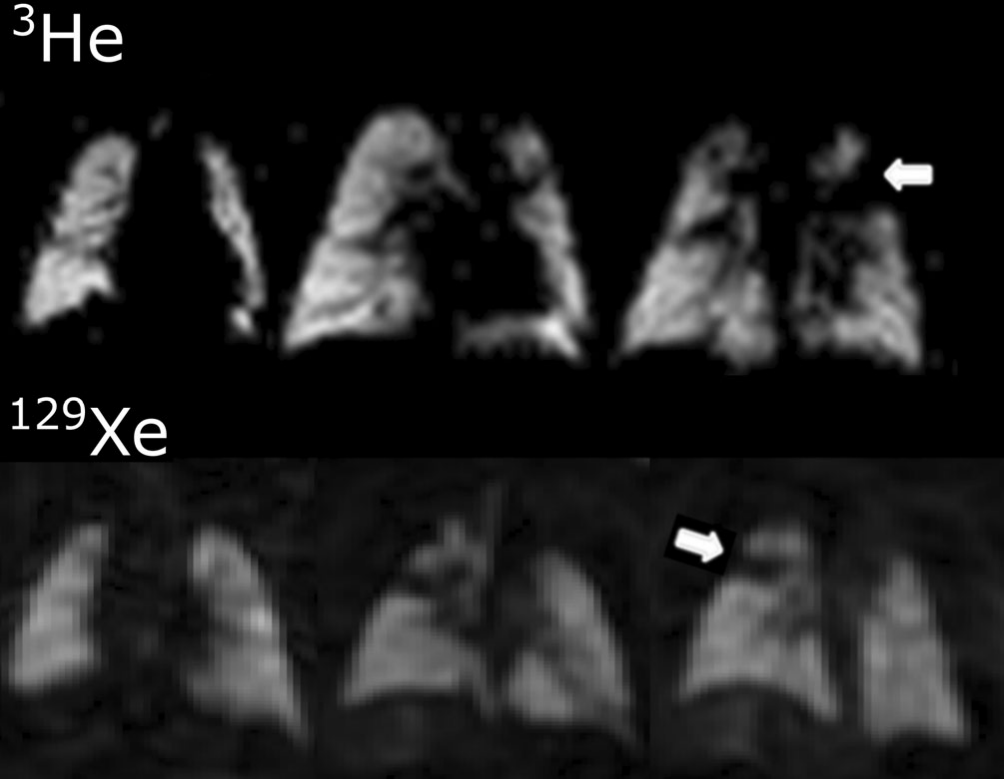
Inhaled hyperpolarized gas images of lung ventilation in a 2‐month‐old infant using ^3^He (top)—reprinted with permission from Reference 102—and a 3‐month‐old infant using ^129^Xe (bottom)—reprinted with permission from Reference 74. Both infants were born prematurely, the latter with confirmed severe bronchopulmonary dysplasia. Arrows indicate major ventilation abnormalities.

The feasibility of hyperpolarized gas lung ventilation imaging in infants between a few months and a few years of age was first shown by Altes et al^102^ using ^3^He gas. Recently, the feasibility of short breath‐hold ^129^Xe imaging for assessment of lung ventilation, microstructure and gas exchange in neonates with BPD has been reported,^74^ providing preliminary evidence of significant ventilation defects and increased ADC indicating altered alveolar microstructure. The latter is in agreement with an ex‐vivo study with hyperpolarized ^3^He, where dramatic alveolar remodeling and enlargement was observed in filamin‐A deficiency—a disease with similar functional presentation to BPD,^103^ and observations in later life as discussed below. Representative ventilation images obtained from Altes et al^102^ and Stewart et al^74^ are shown in Fig. 6.

**FIGURE 7:**
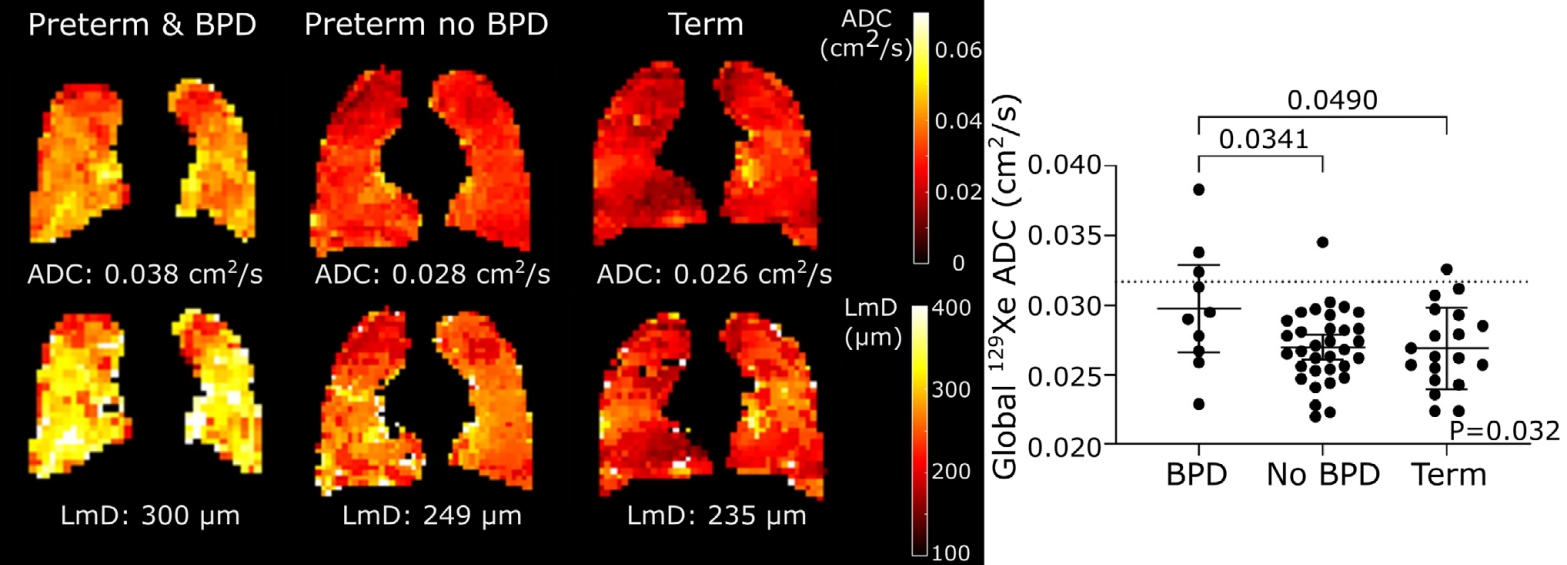
^129^Xe diffusion‐weighted imaging in the lungs of school‐aged children born prematurely with and without bronchopulmonary dysplasia (BPD). Reprinted with permission of the American Thoracic Society.^105^ Copyright © 2024 American Thoracic Society. All rights reserved. The American Journal of Respiratory and Critical Care Medicine is an official journal of the American Thoracic Society. Left: apparent diffusion coefficient (ADC) and lung microstructural dimension (LmD) maps acquired from children born prematurely with and without BPD compared with term born children (global ADC and LmD are quoted). Right: distribution of ^129^Xe ADC values in the three groups showing elevated ADC in children born pre‐term with BPD compared with those born prematurely without BPD and term born children.

BPD in infancy is known to cause lasting lung function deficiency later into childhood. Diffusion‐weighted MRI using both hyperpolarized ^3^He^104^ and ^129^Xe^105^ gas has revealed increased ADC (reflective of increased alveolar size) in school‐aged children with historic BPD‐related pulmonary complications, indicating lung microstructural abnormalities post‐infancy (Fig. 7). An earlier study using ^3^He MRI reported no difference in ADC between schoolchildren with historic BPD and term‐born children,^106^ however, the long diffusion times and non‐imaging nature of the acquisition used were not ideal for measuring alveolar‐restricted diffusion.^107^ Furthermore, prematurity‐related obstructive lung disease was found to be associated with increased ventilation abnormalities on hyperpolarized ^129^Xe MRI,^105^ and both ventilation and perfusion abnormalities on ^1^H FD MRI^108^ in childhood. Further studies of children born prematurely but without a historical diagnosis of BPD are warranted to investigate what insights about lung development and the impact of prematurity can be determined from hyperpolarized ^129^Xe diffusion‐weighted MRI.^106^, ^107^, ^109^

### Cystic Fibrosis

The early detection and sensitive monitoring of lung disease in young children with CF are a prerequisite for optimized care and long‐term outcomes.^110^, ^111^, ^112^, ^113^ Typical characteristics of early CF lung disease are bronchiectasis, wall thickening and mucus plugging.^1^, ^114^ Since wall thickening and mucus plugging are accompanied by an increase in fluid and tissue a.k.a. “plus‐pathologies,” they can significantly improve the conditions for ^1^H MRI, and as such, even pathologies in the peripheral airways can be observed with a similar sensitivity to CT.^115^, ^116^ To date, several studies have demonstrated that chest MRI is sensitive to detect abnormalities in early CF lung disease.^1^, ^117^, ^118^ While wall inflammation demonstrates an obvious contrast enhancement, mucus plugging is especially well visualized by MRI because of its high T_2_ signal, but without contrast enhancement.^119^ Also, bronchial air‐fluid levels in sacculations and consolidations can also be visualized by a high T_2_ signal due to the fluid content.^116^ Pre‐ and post‐contrast T_1_‐weighted imaging can provide complementary information by delineating mucus from the bronchial wall, as mucus does not exhibit enhancement post‐contrast.^116^ In addition to morphological abnormalities, ^1^H MRI can visualize perfusion abnormalities of CF lung disease with pulmonary DCE MRI. As a physiological response to airway obstruction and local hypoxia, delivery of blood to the affected lung region is downregulated (hypoxic pulmonary vasoconstriction).^120^ Thus, perfusion imaging is an excellent tool to monitor functional impairment in CF.

To quantify the pathologies detected by MRI, a chest MRI scoring system was introduced^33^ that consists of a morphology score assessing structural changes (wall thickening/bronchiectasis, mucus plugging, sacculations/abscesses, consolidations; and special findings [mainly pleural reaction]), a perfusion score assessing perfusion abnormalities, and a global score (Table 3). The extent of disease is rated for each lobe as 0 (no abnormality), 1 (<50% of the lobe involved), or 2 (≥50% of the lobe involved). The MRI global score results from the sum of the morphology and perfusion score.

**TABLE 3:** Scoring System for Morpho‐Functional Lung MRI in Cystic Fibrosis

Feature	Right			Left			Maximal Feature/Global Score
	UL	ML	LL	UL	LG	LL	
1. Bronchiectasis/wall thickening	/2	/2	/2	/2	/2	/2	/12
2. Mucus plugging	/2	/2	/2	/2	/2	/2	/12
3. Abscesses/sacculations	/2	/2	/2	/2	/2	/2	/12
4. Consolidation	/2	/2	/2	/2	/2	/2	/12
5. Special findings	/2	/2	/2	/2	/2	/2	/12
6. Perfusion size	/2	/2	/2	/2	/2	/2	/12
*Maximal lobal/global score*	/12	/12	/12	/12	/12	/12	/72

Scoring scheme taken from Reference 33. Each feature is scored from 0 to 2 in each lobe where 0: absent, 1: present in <50% of the lobe, 2: present in >50% of the lobe. UL = upper lobe; ML = middle lobe; LL = lower lobe; LG = lingula.

**FIGURE 8:**
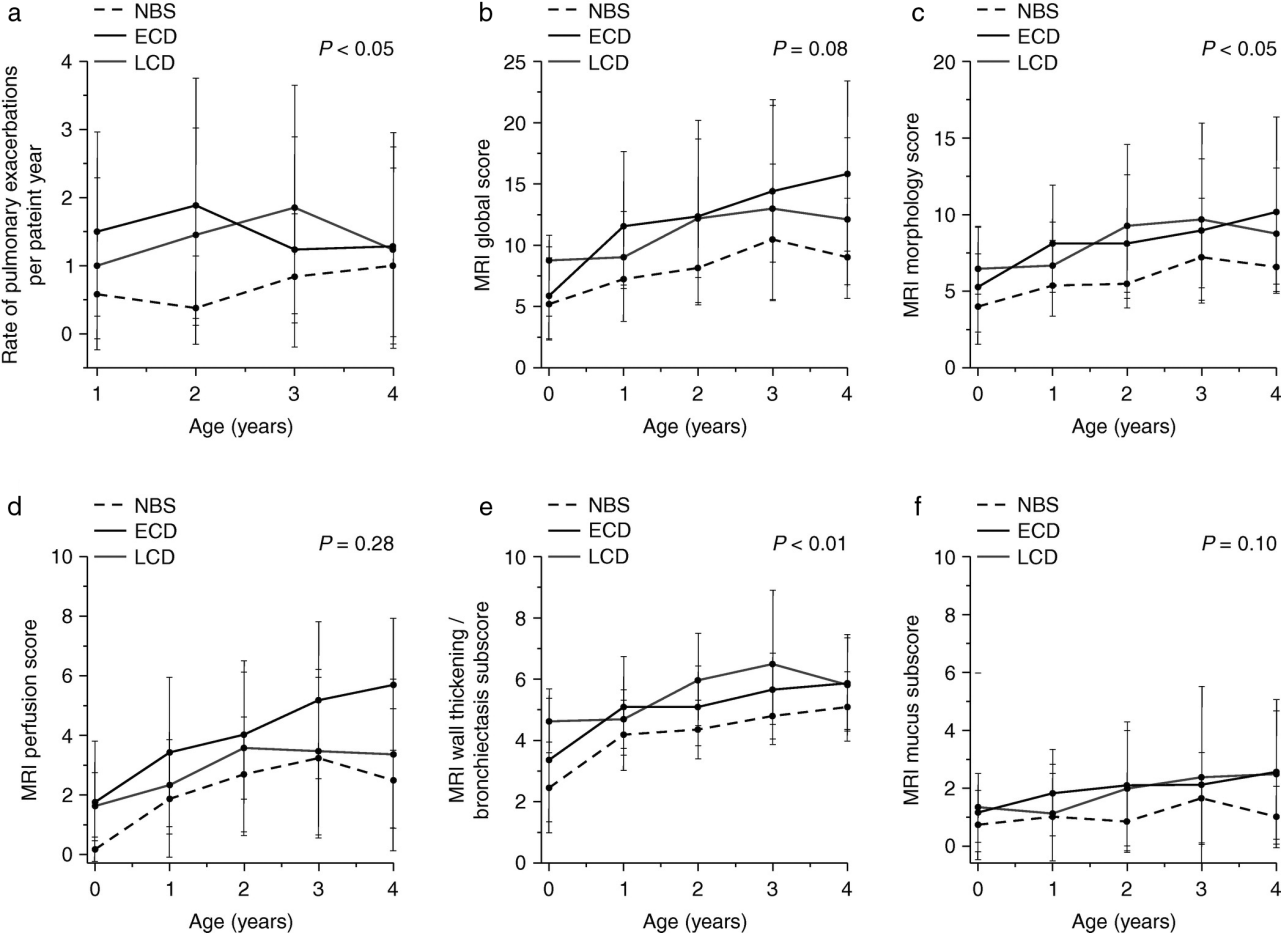
Rate of pulmonary exacerbations (**a**) alongside the time‐course of global (**b**), morphological (**c**), perfusion (**d**), wall‐thickness/bronchiectasis (**e**) and mucus (**f**) MRI scores derived from the scoring system in Table 3 in infants and preschool children with cystic fibrosis over the first few years of life, stratified according to diagnosis method; new‐born screening (NBS), early clinical diagnosis (ECD), late clinical diagnosis (LCD). Reprinted with permission of the American Thoracic Society.^114^ Copyright © 2024 American Thoracic Society. All rights reserved. The American Journal of Respiratory and Critical Care Medicine is an official journal of the American Thoracic Society.

A prospective, longitudinal study in newborns and preschool children with CF (0–4 years, N = 96) demonstrated that MRI with this chest MRI scoring system is sensitive to detect the progression of early lung disease in preschool children with CF.^114^ Moreover, structural lung abnormalities, especially bronchiectasis/wall thickening, detected by MRI were lower throughout infancy and preschool years when the CF diagnosis was established by newborn screening rather than on the basis of clinical symptoms (Fig. 8). These findings demonstrate the importance of early diagnosis in CF to further delay or prevent lung damage in preschool children with CF.^114^ Moreover, an association between the chest MRI score and potential risk factors for the early progression of CF lung disease such as respiratory symptoms, pulmonary exacerbations, anthropometry, and microbiology were found.^114^ In addition to the ability of MRI and the corresponding scoring system for longitudinal diagnostic disease monitoring and detection of disease progression, MRI was found to be sensitive to depict response to antibiotic therapy and disease specific modulator therapy.^1^, ^121^, ^122^

**FIGURE 9:**
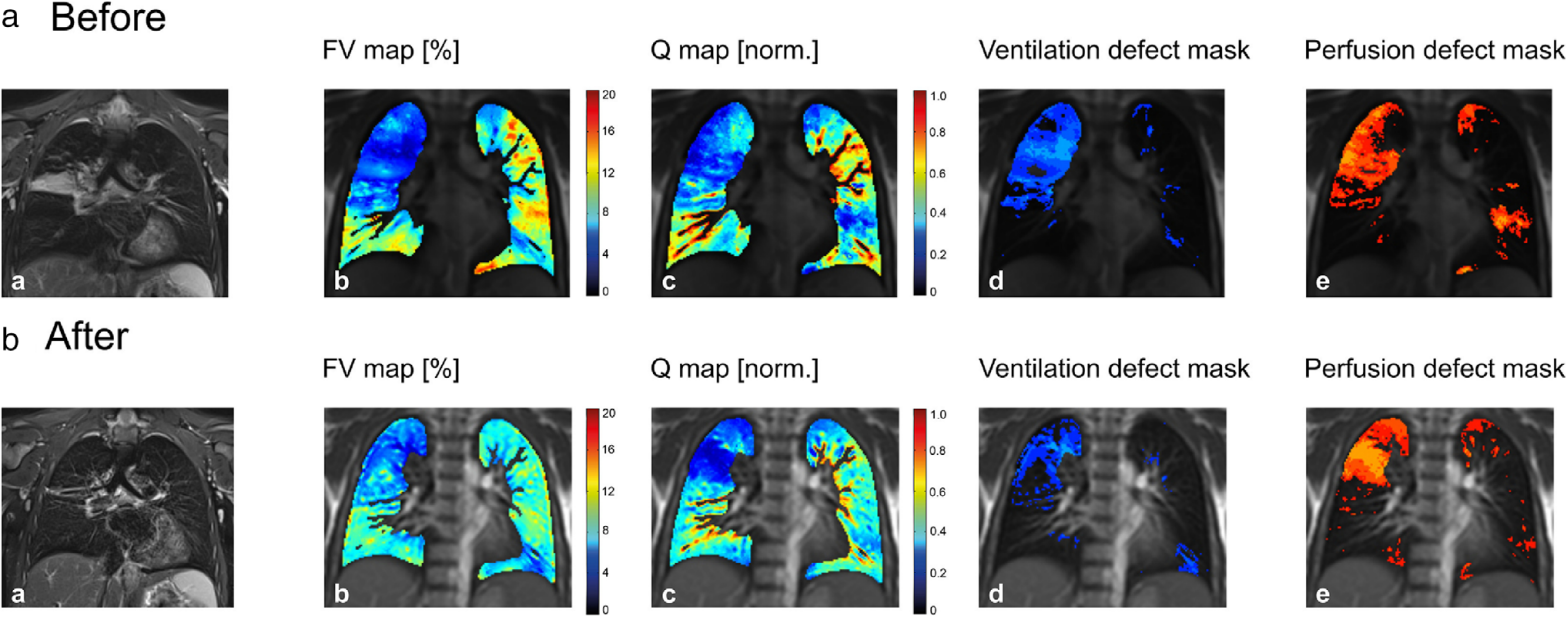
Comparison of pulmonary MRI before and after triple combination therapy in an 11‐year‐old patient with cystic fibrosis. From left to right: morphological (PROPELLER) images, fractional ventilation (FV), perfusion (Q) and ventilation and perfusion defect masks derived from dynamic 2D bSSFP‐based matrix pencil imaging, respectively before (**a**) and after (**b**) the therapy showed partial resolution of atelectasis in the right upper lobe and reduced bronchial wall thickening on morphological imaging, and corresponding improvement of ventilation and reduced ventilation‐perfusion mismatch. Reprinted with permission from Reference 128. bSSFP = balanced steady‐state free precession; PROPELLER = Periodically Rotated Overlapping ParallEL Lines with Enhanced Reconstruction.

In recent years, the feasibility of free‐breathing Fourier Decomposition‐based ^1^H MRI approaches in pediatric CF has been explored.^123^, ^124^ The original FD technique has been validated in adolescents and adults against DCE perfusion visually, and through reader scores,^125^ and recently, in a multi‐site study investigating response to triple combination therapy in children with CF and one or two F508del alleles, one site used PREFUL‐based perfusion in place of contrast‐enhanced perfusion to contribute to the aforementioned MRI score.^121^ PREFUL‐derived ventilation defect percentage was found to decrease after antibiotic treatment for pulmonary exacerbations in school‐aged children with CF.^126^ Similarly, free‐breathing ^1^H MRI with matrix pencil based reconstruction has been used to visualize improvements in both lung ventilation and perfusion metrics in children with CF in response therapy including salbutamol inhalation^127^ and triple combination therapy^128^ (Fig. 9).

**FIGURE 10:**
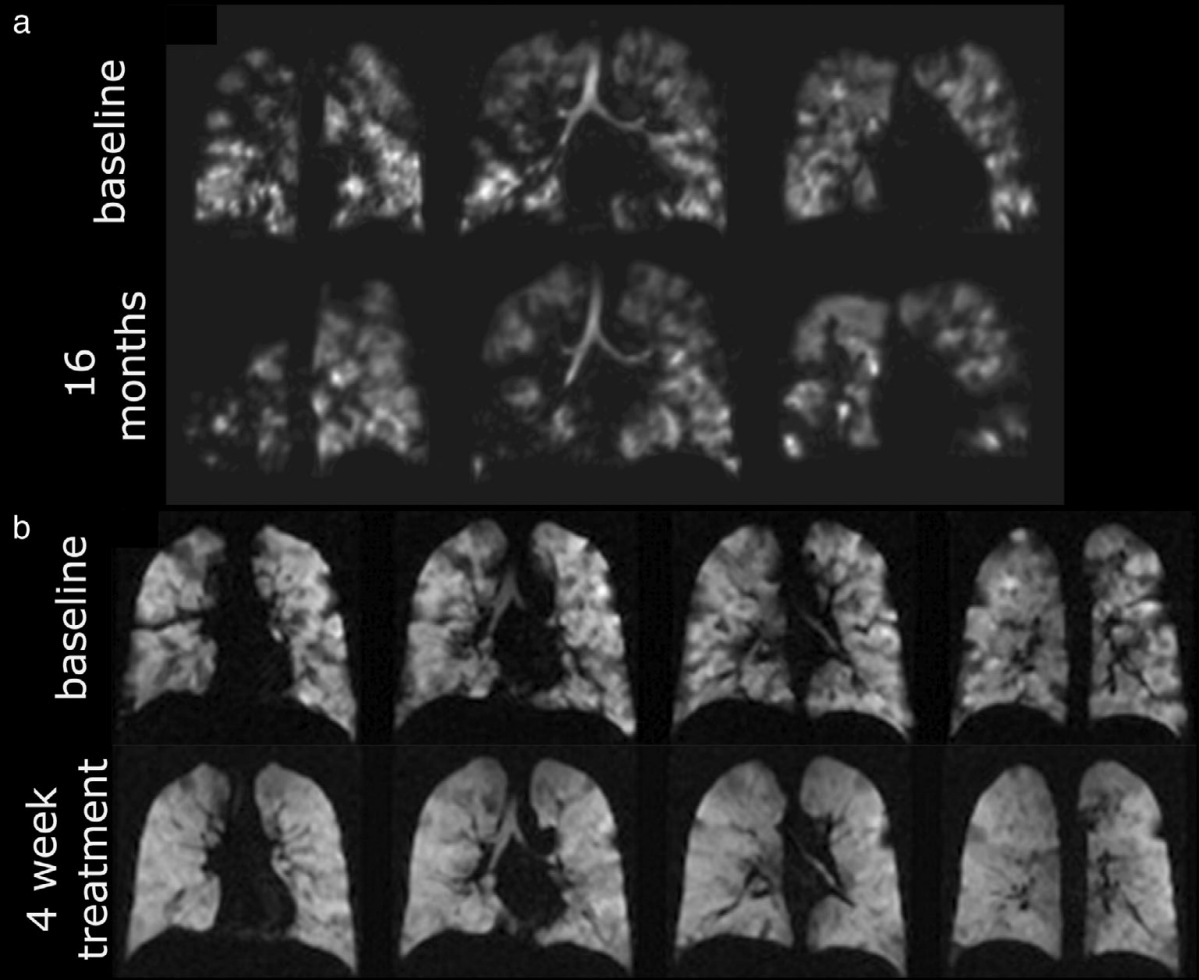
(**a**) Worsening of ventilation heterogeneity (VDP change 16.6% to 27.3%) over 16 months while FEV_1_ *z*‐score and LCI did not change significantly in a pediatric patient with cystic fibrosis (CF) visualized by hyperpolarized ^3^He MRI.^134^ (**b**) Dramatic improvement in ventilation in response to a 4 week course of ivacaftor treatment in an adult with CF visualized by hyperpolarized ^3^He MRI. Reprinted with permission from Reference 138. LCI = lung clearance index; FEV_1_ = forced expiratory volume in 1 second; VDP = ventilation defect percentage.

The assessment of regional ventilation obstruction in children with CF was first explored using dynamic ^3^He gas MRI^129^ and since, hyperpolarized gas MRI ventilation metrics have been shown to correlate strongly with clinical measures including the lung clearance index from multi‐breath washout test.^130^, ^131^ The high sensitivity of hyperpolarized gas MRI to subtle/minor abnormalities in lung ventilation have enabled the detection of sub‐clinical airway obstruction—abnormal VDP where FEV_1_ is normal—in pediatric patients with CF.^132^, ^133^ Furthermore, in longitudinal studies, metrics including VDP and ventilation heterogeneity were reported to reveal sub‐clinical changes at 1‐ or 2‐year follow‐up^130^, ^134^ (Fig. 10a). A key use of this unmatched sensitivity is in the evaluation of therapeutical efficacy,^135^ and this is of great clinical interest with the recent advent of triple combination therapies. In addition to more traditional therapies such as chest physical therapy^136^ and exercise therapy,^137^ strong responses in VDP to ivacaftor therapy in adults with CF^138^ (Fig. 10b) and antibiotics in school‐aged children with CF^139^ have been reported.

**FIGURE 11:**
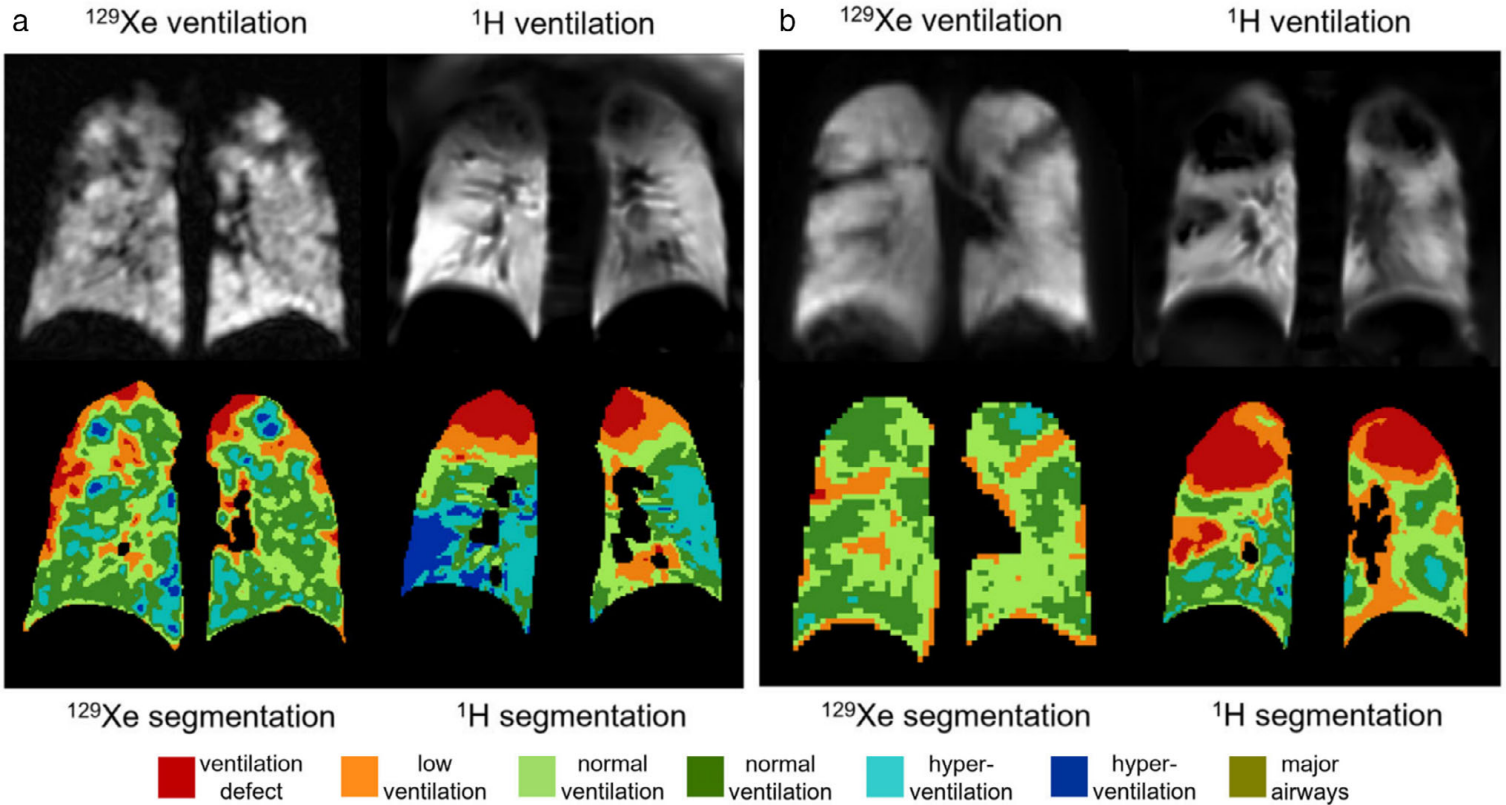
Comparison of breath‐hold ^129^Xe gas‐ and free‐breathing ^1^H PREFUL‐based ventilation maps in pediatric patients with CF, showing a variable level of agreement. Reprinted with permission from Reference 140. (**a**) Patient with mild CF (normal FEV_1_) and (**b**) patient with moderate CF (abnormal FEV_1_). PREFUL = phase‐resolved functional lung; FEV_1_ = forced expiratory volume in 1 second.

Recently, ^129^Xe ventilation MRI has been used as the standard against which to compare ^1^H(PREFUL)‐based MR ventilation metrics in pediatric CF. In a multi‐site study, a strong coarse agreement between the two techniques has been observed with a variable level of intra‐subject disagreement^140^ (Fig. 11), while a separate study reported agreement in stable CF and disagreement in CF patients with exacerbations.^141^ PREFUL and ^129^Xe MRI ventilation measures are reported to have similar repeatability in pediatric CF.^142^ In addition, a multi‐site analysis has been conducted to support the use of ^129^Xe ventilation MRI in multi‐site drug trials, showing excellent agreement in VDP between analysts.^143^

### Pediatric Asthma

Asthma is a highly prevalent chronic respiratory disease characterized by airway inflammation and exacerbations. Most pulmonary MRI studies of asthma to date have focused on adult asthma, however, childhood asthma can present quite differently, and there is a particular need for a personalized management/treatment approach. In school‐aged children with asthma, inhaled ^3^He MRI has been reported to be safe with minimal minor side effects (headache, dizziness).^144^ Ventilation defects are heterogeneous in appearance,^145^, ^146^ and VDP has been found to correlate with clinical metrics including blood eosinophil count.^146^ More recently, ^129^Xe MRI VDP has been shown to correlate with asthma severity and healthcare utilization,^147^ and beyond severity staging, provides a sensitive means to assess bronchodilator response.^148^ Lung MRI is anticipated to find utility in the assessment of novel biologic treatments for pediatric asthma in the immediate future.

### Other Diseases

**FIGURE 12:**
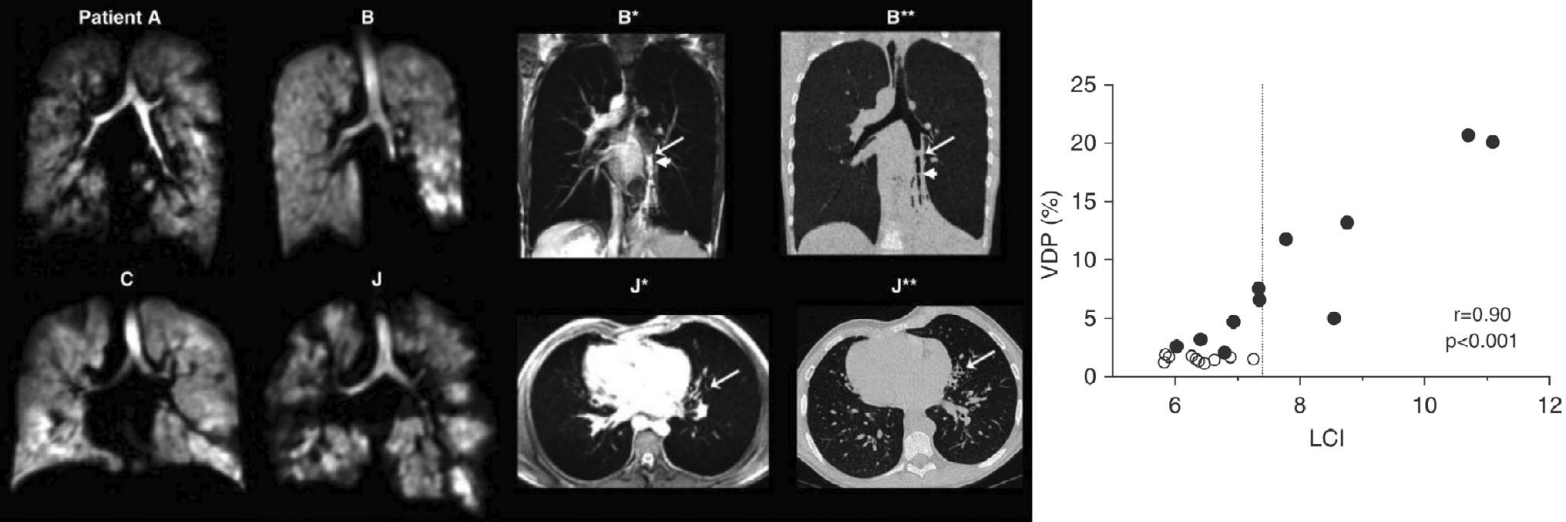
^3^He images of lung ventilation heterogeneity (left) alongside 3D SPGR images of two patients (B* and J*) and computed tomography images (B** and J**) in school‐aged children with primary ciliary dyskinesia (PCD). Reprinted with permission of the American Thoracic Society.^151^ Copyright © 2024 American Thoracic Society. All rights reserved. Annals of the American Thoracic Society is an official journal of the American Thoracic Society. Right: correlation of ventilation defect percentage (VDP) with the lung clearance index (LCI) metric derived from the multi‐breath washout pulmonary function test. SPGR = spoiled gradient recalled echo.

Beyond BPD, CF, and asthma, several other diseases with associated pulmonary morbidity in children have been studied using lung MRI. Primary ciliary dyskinesia (PCD)—a genetic disorder of the cilia causing lung disease in children—shows comparable MRI structural features to CF.^149^ Using matrix‐pencil based structure–function imaging, Nyilas et al found considerable heterogeneity in ventilation and perfusion abnormality and poor correspondence of MRI measures with spirometry in children with PCD.^150^ Single‐site^151^ and multi‐site^152^ studies with hyperpolarized ^3^He and ^129^Xe MRI, respectively, have similarly shown heterogeneity in the presentation of ventilation abnormalities in childhood PCD, and correlation between VDP and lung clearance index (Fig. 12). Willmering et al recently reported reduced ^129^Xe‐MRI derived gas exchange function in pediatric patients after bone marrow transplantation and worsened ventilation heterogeneity in childhood interstitial lung disease (ChILD).^70^ A preliminary study also indicate the similar structural features in ChILD that can be visualized on HASTE and PROPELLER images compared with clinical CT.^153^ Moreover, the utility of ^129^Xe MRI in identifying airway obstruction following hematopoietic stem cell transplant (HSCT) in children has also been explored.^154^ With approval for clinical use of ^3^He and ^129^Xe in Sheffield, UK, and the recent milestone FDA approval of ^129^Xe for ventilation imaging in patients 12 years and above, the utility of hyperpolarized gas MRI in older children in particular is set to expand. We have reported our experience of the impact of hyperpolarized gas MRI in clinical decision making in Sheffield in a recent review.^4^

Take‐home messages/Recommendations:

- Free‐breathing ^1^H UTE imaging may be used in a dedicated NICU‐housed MRI set‐up to inform treatment and management decision making in neonates with BPD
- In CF, ^1^H lung MRI is being used in place of chest x‐ray and CT for first point structural assessment in many centers
- Hyperpolarized ^129^Xe ventilation imaging is sensitive to sub‐clinical disease in pediatric CF
- Diffusion‐weighted and gas‐exchange imaging with ^129^Xe are scientifically interesting but require further evaluation in large pediatric cohorts to establish clinical utility
- Pre‐ and post‐therapy lung MRI is set to play a key role in the short‐ and long‐term assessment of novel therapies in pediatric CF and asthma

## Remaining Challenges

### Breath‐Holds/Sedation

In our experience, children aged 5 years and above can achieve breath‐holds of 5–10 seconds in duration, although this requires coaching by a lung physiologist or physician, which will limit the feasibility more widely. Below this age, free‐breathing methods are the only feasible option. Sedation for MRI is safe and well tolerated,^155^ however, if feasible, imaging without sedation is desirable to lessen the logistical burden of the scan. In infants up to around 6–9 months of age, the feed and swaddle technique allows imaging without sedation.^14^ Between 1 and 4 scan success rates are more variable, although comparable to that of spirometry.^124^ Some centers routinely perform lung MRI under sedation,^86^ and this decision is likely to be made according to local/national guidelines. Recent progress in faster imaging, more sophisticated motion monitoring and correction are all poised to contribute to incrementally improved feasibility of non‐sedation imaging in the near future.

### Research/Work‐in‐Progress/Non‐Vendor Proprietary Sequences/Software

A number of the methods discussed in this article, including radial and spiral ^1^H UTE, are not yet commercially available as products from all the major MRI manufacturers; PREFUL processing of Fourier decomposition MRI requires either the purchase of a software licence or the expertise to implement such processing locally; multi‐nuclear pulse sequences are not yet available on all vendors and/or are not optimized for ^129^Xe by default. Such “research” sequences/methods are currently limited to academic centers, in particular, those with strong research collaborations with the scanner vendors. While standard ^1^H MRI sequences currently provide a comprehensive structural assessment of the lungs, the field is rapidly evolving to evaluate beyond morphology. Through ongoing collaboration between lung imaging centers and scanner vendors, substantial progress toward the integration of more specialized techniques into commercially available products is being made. This progress will lower the hurdle of accessibility to specialized sequences and techniques.

### Accessibility of (Hyperpolarized Gas) Lung MRI

Access to MRI scanners generally remains challenging, especially in developing countries, and as discussed in the following section, low‐field and portable, low‐cost MRI scanners have been developed to address this.^156^, ^157^ Nevertheless, access to hyperpolarized ^129^Xe polarizers and multi‐nuclear hardware and software for MRI scanners is likely to remain restricted to specialized centers due to the significant start‐up costs (up to $500 k for a polarizer, and potentially a similar cost to upgrade a ^1^H‐only scanner to multi‐nuclear capable). A reimbursement model at specialized regional/national referral centers may be achievable in certain developed countries. However, the extra running/prescription costs of ^129^Xe MRI also require consideration; the cost of enriched ^129^Xe is $150 ~ 300 per litre (with up to ~500 mL being used for a single ventilation scan). This reduces to ~$30 per litre for natural abundance xenon, which is similar to the cost of routine spirometry and may provide a more scalable route to widespread clinical use.

### Changing Clinical Guidelines

There is still further work to do to influence national/international guidelines on the use of MRI in pediatric lung disease. MRI is widely accepted at all CF centers in Germany, and by patient organizations.^158^ This is recognized in the recent Fleischner Society guideline paper.^9^ One example of the kind of work the research community needs to do for infant/pediatric lung MRI to influence clinical guidelines/decision‐making more broadly is the work of Adaikalam and colleagues, who showed that the clinical decision‐making process for tracheostomy procedures in infants with BPD can be guided by predictions from lung MRI.^90^ Similar future studies around, eg, MRI‐based characterization of the lung disease phenotypes in patients that most strongly correlate with response to certain clinical therapeutics will help guide clinicians in weighing‐up the cost of therapeutics against their potential benefit in individual patients. Alongside this, we believe that additional research validating functional techniques in the pediatric setting is warranted to allow a widespread clinical application; for hyperpolarized gas MRI and non‐contrast functional imaging such as Matrix Pencil/Fourier Decomposition and PREFUL MRI.

## Future Avenues

In this review, we have summarized the state‐of‐the‐art in lung MRI methods and technology, and the current key areas where lung MRI shows utility in a clinical setting and should find increased application henceforth. In this concluding paragraph, we outline possible research directions for the field that are likely to be a focus of research efforts in the coming years. First, the recent resurgence in low‐field MRI has potentially dramatic consequences for the lung MRI field, given the SNR improvement for ^1^H imaging of the lung parenchyma.^159^ The reduced footprint is also attractive in terms of improved sustainability and increased accessibility, which may allow scanners to be placed closer to pediatric/neonatal wards. The advent of niche, cheap and portable low‐field systems are promising for point‐of‐care brain imaging^160^ but the attainable image quality in the lung is yet to be evaluated. Second, as MRI acquisition methods for the lung have matured, the focus of present research is showing a trend toward developing more robust and informative analyses, and the use of artificial intelligence (AI) in lung image reconstruction,^161^, ^162^ segmentation,^163^, ^164^ functional mapping and registration.^165^ To date the applications of AI in lung MRI have been limited to studies in adults, and this largely reflects the weighting of the training of these models toward images acquired in adults, and the larger “market” in terms of numbers of patients living with lung disease. In a recent review of the status of AI in chest CT and x‐ray, although >20 AI products available for clinical use were reviewed, very few of these showed any application for pediatric imaging,^166^ and we may expect a similar situation with MRI AI products. The ethical challenges governing the use of lung images from children to train AI models and how this differs from the situation in adults is beyond the scope of the current article,^167^ but in the near future we would expect tailored AI for accelerated imaging and reconstruction of motion‐artifact free images when AI models are trained with high SNR gated images in children. Furthermore, AI‐driven protocolling should allow for rapid turnaround of pediatric scans, crucial for pre‐school children in particular who may have limited compliance in the scanner. Finally, beyond lung imaging, MRI of the major airways has been relatively under‐explored outside of the neonatal population,^90^ but has the potential to inform treatment decisions—eg, reducing the number of invasive bronchoscopic procedures—in pediatric airway disorders such as obstructive sleep apnea, and can be acquired relatively easily in the same session as lung images.
